# Pathology Assessments of Multiple Organs in Fatal COVID-19 in Intensive Care Unit vs. Non-intensive Care Unit Patients

**DOI:** 10.3389/fmed.2022.837258

**Published:** 2022-04-25

**Authors:** Yoann Zerbib, Nelly Guilain, Sébastien Eymieux, Rustem Uzbekov, Sandrine Castelain, Emmanuelle Blanchard, Catherine François, Denis Chatelain, Clément Brault, Julien Maizel, Philippe Roingeard, Michel Slama

**Affiliations:** ^1^Intensive Care Unit, Amiens Picardie University Hospital, Amiens, France; ^2^Department of Pathology, Amiens Picardie University Hospital, Amiens, France; ^3^INSERM U1259 MAVIVH, Université de Tours and CHRU de Tours, Tours, France; ^4^Plate-Forme IBiSA de Microscopie Electronique, Université de Tours and CHRU de Tours, Tours, France; ^5^Faculty of Bioengineering and Bioinformatics, Moscow State University, Moscow, Russia; ^6^Department of Virology, Amiens Picardie University Hospital, Amiens, France; ^7^Agents Infectieux, Résistance et Chimiothérapie, Research Unit, AGIR UR4294, University of Picardie Jules Verne, Amiens, France

**Keywords:** lung pathology, ARDS, ICU, SARS-CoV-2, COVID-19

## Abstract

**Purpose:**

The objective of the present study was to provide a detailed histopathological description of fatal coronavirus disease 2019 (COVID 19), and compare the lesions in Intensive Care Unit (ICU) and non-ICU patients.

**Methods:**

In this prospective study we included adult patients who died in hospital after presenting with confirmed COVID-19. Multiorgan biopsies were performed. Data generated with light microscopy, transmission electron microscopy (TEM) and RT-PCR assays were reviewed.

**Results:**

20 patients were enrolled in the study and the main pulmonary finding was alveolar damage, which was focal in 11 patients and diffuse in 8 patients. Chronic fibrotic and inflammatory lesions were observed in 18 cases, with acute inflammatory lesions in 12 cases. Diffuse lesions, collapsed alveoli and dystrophic pneumocytes were more frequent in the ICU group (62.5%, vs. 25%; 63%, vs. 55%; 87.5%, vs. 54%). Acute lesions (82%, vs. 37.5%; *p* = 0.07) with neutrophilic alveolitis (63.6% vs. 0%, respectively; *p* = 0.01) were observed more frequently in the non-ICU group. Viral RNA was detected in 12 lung biopsies (60%) up to 56 days after disease upset. TEM detected viral particles in the lung and kidney biopsy samples up to 27 days after disease upset. Furthermore, abundant networks of double-membrane vesicles (DMVs, a hallmark of viral replication) were observed in proximal tubular epithelial cells.

**Conclusion:**

Lung injury was different in ICU and non-ICU patients. Extrapulmonary damage consisting in kidney and myocardial injury were more frequent in ICU patients. Our TEM experiments provided the first description of SARS-CoV-2-induced DMVs in kidney biopsy samples—a sign of intense viral replication in this organ.

## Introduction

In December 2019, severe acute respiratory syndrome coronavirus 2 (SARS-CoV-2) emerged in Wuhan, China. It is now responsible for an ongoing pandemic of coronavirus disease 2019 (COVID-19) (1, 2). The scientific community responded rapidly by determining the virus’s characteristics, understanding its mode of transmission, identifying clinical presentations, and testing possible treatments. The literature data now provide a precise overview of the SARS-CoV-2 infection and its clinical consequences (3). The main clinical manifestation of symptomatic and severe form is acute respiratory failure, which can require admission to an intensive care unit (ICU) and then mechanical ventilation (4–6). Computed tomographic imaging typically shows a “ground glass” pattern in the peripheral areas of the lung. The overall clinical and radiographic presentation meets the Berlin criteria for acute respiratory distress syndrome (ARDS), of which diffuse alveolar damage (DAD) is a classical but non-specific sign (7–9). Large vessel thrombosis and vascular changes are also important features of COVID-19 (8, 10). A growing body of evidence indicates that SARS-CoV-2 can affect organs other than the lungs—leading to multiple organ failure and death (11). However, histological data and autopsy findings on this topic are scarce. Although several histological studies of COVID-19 have been published, few of these looked at biopsies from a large number of organs and sought to detect SARS-CoV-2 virions in the tissues (11, 12).

Hence, the objective of the present study was to provide a detailed histological description of fatal COVID 19 and compare the lesions in patients admitted to ICU with those not admitted to ICU.

## Materials and Methods

### Study Design and Population

We performed a prospective, single-center study in a teaching hospital (Amiens Picardie University Hospital, Amiens, France). From March to May 2020, adult patients who died in hospital after presenting with confirmed COVID-19 were screened for inclusion in the study. Cases of COVID-19 were defined according to the World Health Organization classification. After having received verbal consent from family members and checked the French national register of organ donation refusals, we performed surgical biopsies of the lungs, heart, and muscle tissue and needle biopsies of the kidneys and liver. The study was approved by the French Ministry of Health (reference number: PFS 20-010).

### Data Collection

We collected data on demographics (including age and gender), the main comorbidities, the initial clinical presentation, the date of symptom onset, and standard laboratory test results on admission. The timeline of organ failure was determined from the clinical signs and biomarker assay results.

### Procedure for Surgical and Needle Biopsies

A two-centimeter incision was made in the fourth intercostal space on the midaxillary line. After removal of the pectoral muscle, we incised the pleura and the pericardium and took surgical biopsies of the lungs and the myocardium. If the lungs were not homogeneously affected, we sampled the most obviously diseased area. We then used a 14-gauge biopsy needle to perform ultrasound-guided percutaneous liver and kidney biopsies. All samples were prepared for pathological assessment and a RT-PCR assay for SARS-CoV-2. For some samples, the tissue ultrastructure was analyzed using transmission electron microscopy (TEM).

### Pathology Assessments

The post-mortem biopsy samples were fixed in buffered 4% formaldehyde for 24–48 h and embedded in paraffin. Sections (thickness: 3 μm) were prepared with a microtome and then stained with hematoxylin-eosin-saffron reagent. Furthermore, liver sections were stained with Masson’s trichrome and Perls Prussian Blue, kidney sections were stained with Masson’s trichrome, periodic acid Schiff (PAS), Gomori and Congo Red, and lung sections were stained with PAS, Grocott silver stain, and Gram stain.

All lung biopsies were immunohistochemically stained with antibodies against CD3 (Dako, Glostrup, Denmark 1/50), CD4 (Ventana, Tucson Arizona United States, prediluted), CD8 (Novocastra, Newcastle, United Kingdom, 1/20), CD163 (Ventana, Tucson Arizona United States, prediluted), and factor VIII (Dako, Glostrup, Denmark 1/100) and cytomegalovirus (Dako, Glostrup, Denmark, prediluted). The slides were reviewed by two pathologists, both of whom were unaware of the patients’ clinical status. On the basis of these findings, the pathologists suggested the most likely cause of death. The suggested cause was then compared with the clinical data.

### Molecular Detection of SARS-CoV-2 RNA

RNA was extracted from nasopharyngeal swabs and post-mortem biopsies, using the NucliSENS^®^ easyMAG^®^ technique (bioMérieux, Craponne, France) (13). Prior to extraction, 10 mg of biopsy tissue were lysed in 200 μl of lysis buffer and incubated for 30 min at 56°C. As described previously, the 25 μl reaction volume comprised 5 μl of RNA, 12.5 μl of the 2x reaction buffer provided with the Superscript III one-step RT-PCR system with Platinum Taq polymerase (Invitrogen), 1 μl of reverse transcriptase/Taq mixture from the kit, 0.4 μl of a 50 mM magnesium sulfate solution, the pair of primers and the probe for the IP2 and IP4 regions of the RdRp gene (14). Incubation at 55°C for 20 min (for reverse transcription) and then at 95°C for 3 min was followed by 50 thermal cycles of 95°C for 15 s and 58°C for 30 s.

### Ultrastructural Analysis, Using Transmission Electron Microscopy

When both lung and kidney biopsies were available for a given patient, we performed an ultrastructure analysis of each organ with TEM. The post-mortem lung and kidney biopsies were fixed by incubation for 24 h in 1% glutaraldehyde/4% paraformaldehyde (Sigma, St Louis, MO, United States) in 0.1 M phosphate buffer (pH 7.2). The samples were washed in phosphate-buffered saline, post-fixed by incubation with 2% osmium tetroxide (Agar Scientific, Stansted, United Kingdom) for 1 h, fully dehydrated (using a graded series of ethanol solutions and propylene oxide), impregnated with a 1:1 mixture of propylene oxide/Epon resin (Sigma) and left overnight in pure resin. The samples were then embedded in Epon resin, which was allowed to polymerize for 48 h at 60°C. Ultrathin (90 nm) sections were prepared with a Leica EM UC7 ultramicrotome (Wetzlar, Germany), stained with 2% uranyl acetate (Agar Scientific) and 5% lead citrate (Sigma), and observed with TEM (JEOL 1400, JEOL Ltd., Tokyo, Japan).

### Statistical Analysis

Continuous variables were expressed as median and interquartile range (25th and 75th percentiles) and were compared by using the Mann–Whitney *U* test. Categorical variables were expressed as numbers and percentages and were compared by the chi-square test or the Fisher exact test as appropriate. In order to analyze differences in pathology as a function of disease duration, we divided the cohort into 2 subgroups based on median disease duration.

## Results

### Clinical Data

During the 3-month study period, post-mortem biopsies of at least two organs were obtained from 20 non-consecutive patients {median age [interquartile range (IQR)] age: 75.5 (66.8–84.3)}. Given the patients’ age, major comorbidities (and especially cardiovascular comorbidities) were highly prevalent (Table 1). The signs and symptoms most frequently encountered at admission were dyspnea, cough and fever. Neurological symptoms (confusion and anosmia) and gastrointestinal signs and symptoms (nausea, vomiting, and diarrhea) were less frequent. Laboratory tests revealed profound lymphopenia in 7 patients (35%). Patients had high serum levels of the inflammatory marker C-reactive protein [mean (IQR): 150 (65.5–214.5) mg/l]. Ten patients (50%) developed acute kidney failure, as evidenced by an elevated blood creatinine level. Seven patients (35%) had elevated troponins. Bacterial co-infection was suspected in 10 patients (50%). Eight patients (40%) were admitted to the ICU and required mechanical ventilation (Tables 1, 2).

**TABLE 1:** Patient characteristics, comorbidities, and outcomes.

Variable	Value
Sex: male/female ratio	14/6
Age, years [median (IQR)]	75.5 [66.8–84.3]
Comorbidities, *n* (%):	
Active smoking	5 (25%)
Chronic obstructive pulmonary disease	4 (20%)
Hypertension	14 (70%)
Cardiovascular disease	8 (40%)
diabetes	8 (40%)
Chronic kidney disease	7 (35%)
Chronic liver disease	1 (5%)
Cancer	5
Body mass index (kg/m^2^)	26.4 [24.5–29.4]
Time between disease onset and hospital admission (d)	3 [0.75–10.25]
Disease duration (time between disease onset and death) (d)	18 [10–32]
Specific treatments received:	
Lopinavir	2 (10%)
Steroids	5 (25%)
Hydroxychloroquine	3 (15%)
Azithromycin	9 (45%)
Outcomes:	
Length of hospital stay	13.5 [6–24]
Admission to the ICU	8 (40%)
Respiratory support	8 (40%)

**TABLE 2:** Patient characteristics, symptoms, organ failure and outcomes.

	Age Years	Sex	Comorbidities	Presenting symptoms	Time between symptom onset and hospital admission	Disease duration	Kidney damage	Myocardial damage	Admission to the ICU	Post-mortem SARS-CoV-2 RNA detection (PCR)
1	66	Female	*Smoker, COPD, lung cancer*	*Fever, dyspnea, cough, chest pain, fatigue*	1	10	No	No	No	Lung
2	73	Male	None	Fever, dyspnea, confusion	24	36	Yes	No	No	Unknown
3	83	Male	*COPD, diffuse interstitial lung disease, hypertension, atrial fibrillation, ischemic cardiomyopathy*	*Nausea and vomiting,*	0	15	No	Yes	No	Lung
4	93	Male	*Hypertension, chronic kidney disease*	*Fever, dyspnea, diarrhea*	14	18	Yes	No	No	Lung
5	78	Female	*Hypertension, ischemic cardiomyopathy, cancer*	*Dyspnea, cough*	11	25	No	No	No	No
6	87	Female	*Pulmonary embolism, restrictive lung disease, hypertension, ischemic cardiomyopathy, diabetes, cancer*	*Dyspnea, cough, diarrhea, confusion, anosmia, dysgeusia*	3	9	No	Yes	No	No
7	90	Female	*Hypertension, chronic kidney disease, diabetes*	*Dyspnea*	3	9	Yes	No	No	No
8	62	Male	*Hypertension, chronic kidney disease,*	*Fever, dyspnea*	12	39	Yes	Yes	Yes	Lung
9	71	Male	*Smoker, hypertension, ischemic cardiomyopathy, chronic kidney disease, diabetes*	*Dyspnea, cough, confusion,*	3	8	Yes	Yes	No	Lung
10	59	Male	*Diabetes, chronic liver disease*	*Fever, dyspnea*	0	17	No	No	Yes	Lung
11	72	Male	*Cardiovascular disease,*	*Fever,*	1	2	-	-	No	Lung
12	67	Male	*Hypertension, chronic kidney disease, diabetes*	*Dyspnea*	0	27	Yes	No	Yes	Lung
13	82	Male	*Smoker, hypertension, ischemic cardiomyopathy, chronic kidney disease,*	*Dyspnea*	14	30	Yes	No	Yes	Lung
14	84	Male	*Hypertension, ischemic cardiomyopathy, restrictive lung disease, diabetes*	*Dyspnea*	4	10	Yes	Yes	Yes	Lung
15	85	Male	*Ischemic cardiomyopathy, chronic kidney disease,*	*Dyspnea, cough, chest pain*	0	23	Yes	No	No	Lung, Kidney
16	64	Male	*Cancer,*	*Fever, cough,*	10	52	Yes	Yes	Yes	Lung, Heart
17	93	Male	*Hypertension, diabetes*		1	1	No	No	No	No
18	61	Male	*Hypertension, diabetes*	*Dyspnea, nausea and vomiting,*	4	56	Yes	Yes	Yes	Unknown
19	80	Female	*Hypertension,*	*Fever*	7	55	No	No	No	Lung
20	73	Female	*Hypertension, ischemic and rhythmic cardiomyopathy, pulmonary embolism*	*Fever, dyspnea*	3	16	No	Yes	Yes	No

### Pathology Assessments

Post-mortem biopsies were taken from the following organs: the heart (*n* = 20 patients), lungs (*n* = 20), liver (*n* = 20), kidneys (*n* = 17), striated muscle (*n* = 16), thyroid (*n* = 8), and adipose tissue (*n* = 4).

#### Lung Biopsies

**FIGURE 1:**
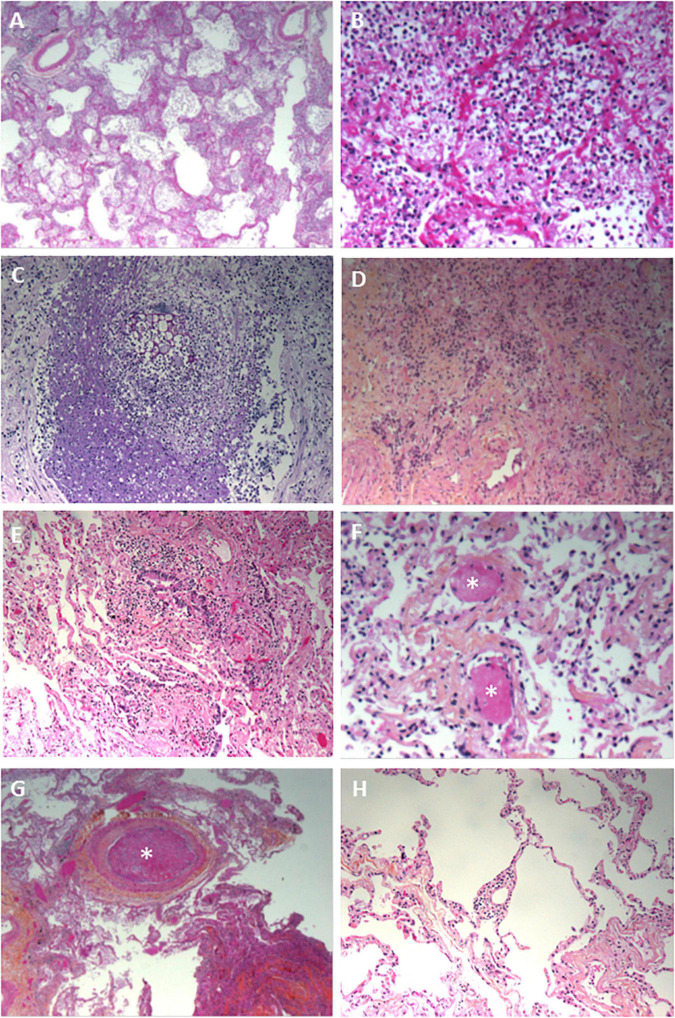
Pathology findings for the lungs in deceased patients with COVID-19. **(A)** Fibrinous alveolitis; hematoxylin-eosin-saffron × 100. **(B)** Suppurative alveolitis with alveoli full of neutrophils and fibrin; hematoxylin-eosin-saffron, × 400. **(C)** Collapsed alveoli with thickened fibrous septa and an inflammatory infiltrate (lymphocytes and macrophages); hematoxylin-eosin-saffron, × 100. **(D)** Aspiration pneumonia with a foreign body and neutrophils in the lumen of a bronchiole; hematoxylin-eosin-saffron, × 400. **(E)** Bronchiolitis, lymphocytic infiltrate surrounding a bronchiole; hematoxylin-eosin-saffron, × 100. **(F)** Microthrombi in lung capillaries; hematoxylin-eosin-saffron, × 400. **(G)** Pulmonary embolism with a fibrinous thrombi in the lumen of a pulmonary artery; hematoxylin-eosin-saffron, × 100. **(H)** Lymphocytic vasculitis, with lymphocytic infiltrate surrounding a small pulmonary vessel; hematoxylin-eosin-saffron, ×100.

Nineteen of the 20 lung biopsies (95%) were informative. The lung damage was focal in 11 cases and diffuse in 8 cases. Chronic fibro-inflammatory lesions were present in 18 cases, with acute inflammatory lesions seen in 12 cases (Figures 1A,B). Aspiration pneumonitis was seen in 2 cases, with fibrin, neutrophils and macrophages in numerous alveoli and with foreign bodies in some bronchioles and alveoli (Figure 1C). Interstitial organized pneumopathy was seen in 18 cases, with enlarged septa containing a few mononuclear cells and muscle bundles. Intra-alveolar buds of granulation tissue (consisting of fibroblasts, myofibroblasts and fibrous or loose connective tissue) were observed in 12 cases (Figure 1D). A few fibrin deposits in some alveoli were seen in 5 cases. Foci of CD3-, CD8- and CD4-positive T lymphocytes and foci of CD163-positive macrophages were seen in enlarged intra-alveolar septa in 18 cases. In 2 cases, we observed bronchiolitis with a dense inflammatory infiltrate (small T lymphocytes and macrophages) around the bronchioles (Figure 1E). The epithelial cells lining the bronchioles were normal. Foci of CD163-positive macrophages in some alveoli were found in all 19 cases. There were a few multinuclear macrophages in 5 cases. No factor-VIII-positive megakaryocytes were observed. Dystrophic pneumocytes with enlarged nuclei were seen in 16 cases. Nuclear eosinophilic inclusions in the pneumocytes were found in only one case and were negative when stained for cytomegalovirus. Fibrinous thrombi in small vessels were seen in 12 cases (Figures 1F,G). Lymphocytic infiltrates around vessels were seen in 8 cases (Figure 1H). Endothelial cell desquamation was seen in one case, although the tissue had been somewhat altered by post-mortem autolysis. Dystrophic vessels with enlarged fibrous intima and media were observed in 12 cases. Amyloid deposits were present in the small lung vessel walls in 2 cases and bone marrow embolism was present in 2 cases. Viral RNA was detected in 12 lung biopsies (60%). No fungi or bacteria were detected (using special stains) in any of the cases.

**FIGURE 2:**
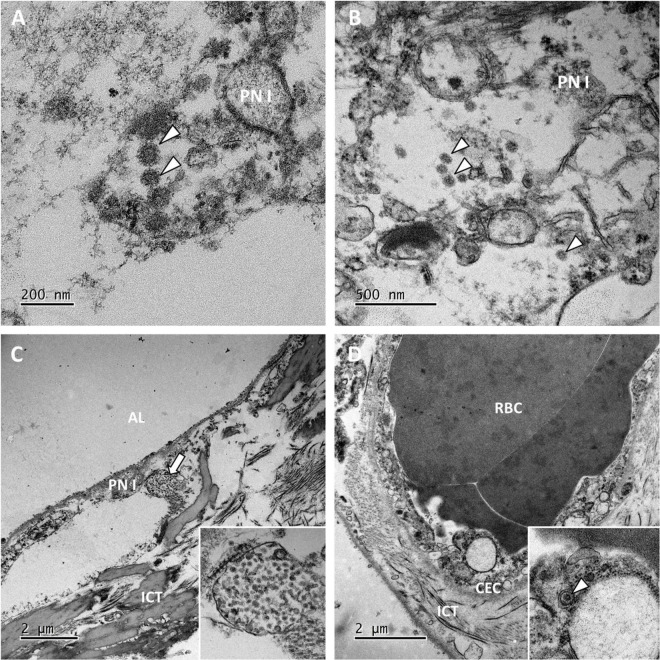
Ultrastructural findings in the lungs. **(A,B)** Viral particles harboring the typical crown of spikes projection (white arrowheads) were present in the cytosol of lytic type I pneumocytes (PN I) in patients 11 and 12. **(C)** In the pneumocytes from patient 12, viral particles also accumulated in large vacuoles (the white arrow; see the inset for a higher magnification image). AL, alveolar lumen; ICT, interstitial connective tissue. **(D)** Lung capillaries were often obstructed with red blood cells (RBCs). In patient 10, capillary endothelial cells (CECs) occasionally contained a few isolated viral particles in small vesicles (the white arrowhead in the inset).

Transmission electron microscopy ultrastructure analysis was performed on samples from 6 patients (patients 3, 5, 10, 12 and 14). In all cases, the lung parenchyma showed alveolar damage, with detached and lytic pneumocytes in the lumen. Enveloped particles harboring spike projections (similar to those observed in cells cultured *in vitro* with SARS-CoV-2) were observed in 3 biopsies (patients 10, 11 and 12) where disease duration were 17, 2 and 27 days respectively (Figures 2A,B) (15). These viral particles were quite homogeneous in size and shape. They measured 80–90 nm without their spikes (110–120 nm with spikes) and presented round to oval shapes. Cross-section through their nucleocapsid showed an electron dense black dot inside the particle. When particle morphology was sufficiently preserved, they harbored five to ten spikes visible on the outside on one section. Viral particles were detected mostly in type I pneumocytes (Figure 2C) and very rarely in type II pneumocytes. The particles formed small aggregates in the cytosol and (although less frequently) in large intracellular vacuoles. Some of these large vacuoles included a very large number of viral particles. The lung capillary lumens were obstructed with erythrocytes; this was consistent with the blood stasis lesions observed in COVID-19. For one patient, a small number of viral particles were observed in the cytoplasm of capillary endothelial cells (Figure 2D). No viral particles were found in samples from the remaining 3 patients.

#### Kidney Biopsies

Only 12 kidney biopsies (70%) were informative. The renal tissue was normal in 4 cases; post-mortem necrosis of the epithelial cells lining the proximal and distal tubes was considered to be normal. In 5 cases, the small renal arteries showed severe arteriosclerotic lesions, with thickened, fibrosed intima and media. In 6 cases, we observed small foci of lymphocytes in the interstitium but considered these to be non-specific signs of interstitial nephritis. In 3 cases, collagen deposits in a few glomeruli (consistent with a diagnosis of focal and segmental hyalinosis) were seen. In one case, we found a large number of neutrophilic micro-abscesses in the interstitium around the tubules and glomeruli; this was consistent with a diagnosis of acute pyelonephritis. Neither amyloid deposits nor crescentic glomerulopathies were seen in any of the cases.

**FIGURE 3:**
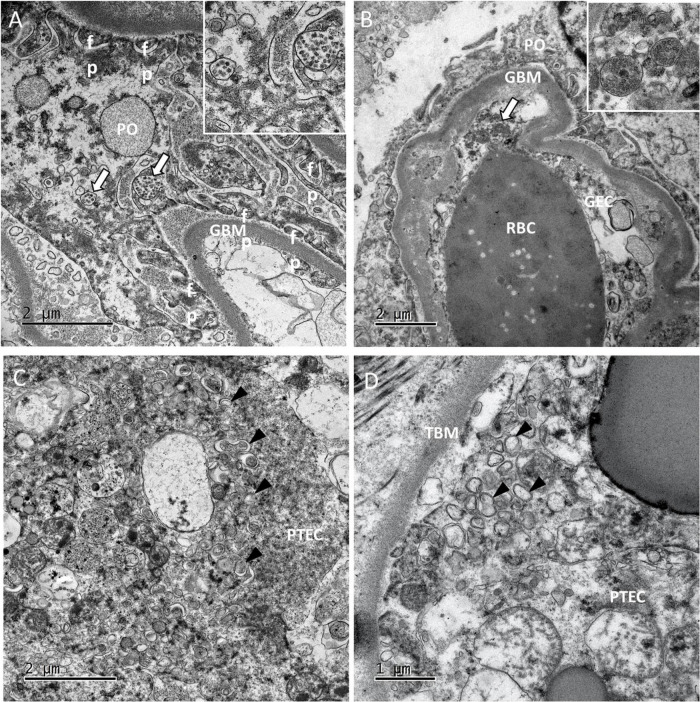
Ultrastructural findings in the kidneys. **(A)** In glomeruli of patient 5, large numbers of viral particles in intracellular vacuoles (white arrows; see the inset for a higher magnification image) were found in podocytes (PO). GBM, glomerular basement membrane; fp, foot process. **(B)** In patient 10, glomerular capillaries showed signs of blood stasis. We also noticed vacuoles filled with smooth viral particles (white arrow) in the cytoplasm of glomerular endothelial cells (GECs). **(C,D)** In the tubules of patient 10 and 11, proximal tubular epithelial cells (PTECs) contained an abundant network of double-membrane vesicles (DMVs; black arrowheads), a marker of SARS-CoV-2 replication. TBM, tubular basement membrane. The DMVs are easily recognizable, with an inner membrane and an outer membrane [white arrowheads in the insets in panels **(C,D)**].

Viral particles were revealed by TEM in 2 patients. In the glomeruli, the particles were present mainly in the podocytes (Figure 3A) and more rarely in capillary endothelial cells (Figure 3B). Viral particles were also found in proximal tubular epithelial cells. These particles shared the same characteristics with those previously described in the lung biopsies (16). As seen in the lung capillaries, signs of blood stasis were also present in the glomerular capillaries. In one case in which viral particles were not found, some proximal tubular epithelial cells contained an abundant network of double-membrane vesicles (DMVs, an ultrastructural characteristic of SARS-CoV-2 infected cells) (15) (Figure 3C). These double-membrane organelles are compatible in size (200–300 nm) and structures with those observed in SARS-CoV-2 infected cell lines (15, 17). Contrary to cellular autophagosomes, which are often very heterogeneous in size and contain cellular structures such as membrane whorls, these elements were quite homogenous in size and morphology. It has been suggested that in poliovirus and hepatitis C virus, these DMVs anchor the viral replication complexes and thus conceal viral RNA from innate immune sensors (18). Although cytoplasmic structures can sometimes be misinterpreted as viral particles, the presence of large numbers of these distinctive DMVs suggested strongly that SARS-CoV-2 had replicated in the patients’ proximal tubular epithelial cells *in vivo* (19–21). Smaller number of DMVs were also observed in tubular cells from one patient in whom viral particles had also been found (Figure 3D) even after a prolonged disease duration (17 days). No viral particles or DMVs were detected in the kidney biopsies from the other 3 patients. Two of the three patients with viral particles and/or DMVs in the kidney also had viral particles in the lung biopsies.

#### Myocardial Biopsies

**FIGURE 4:**
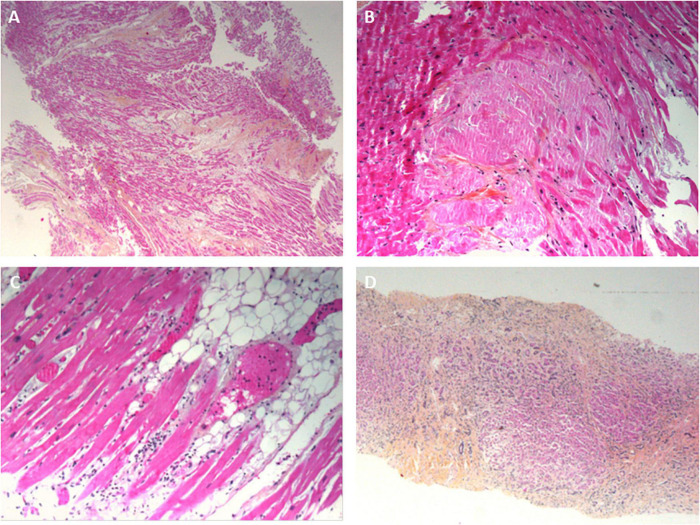
Pathology findings for other organs in deceased patients with COVID-19. **(A)** Ischemic cardiomyopathy, with a fibrous scar in the myocardium; hematoxylin-eosin-saffron, ×100. **(B)** Cardiac amyloidosis, with eosinophilic deposits of amyloidosis around the myocytes; hematoxylin-eosin-saffron, ×400. **(C)** A myocardial biopsy showing a mild inflammatory infiltrate around the myocytes. **(D)** Liver cirrhosis: extensive fibrosis surrounding micronodules composed of small hepatocytes. Numerous cholangioles and mononuclear inflammatory infiltrates in the fibrous bands; hematoxylin-eosin-saffron, ×100.

All the myocardial biopsies (100%) were informative. The myocardial tissue was normal in 9 cases (45%); interstitial edema and post-mortem dystrophic cytoplasmic or nuclear alterations of the myocytes were considered to be normal. In 9 cases (45%), fibrotic deposits of varying abundance were observed in the interstitial tissue. The deposits included macrophages in one case (consistent with a diagnosis of chronic ischemic cardiopathy) and a few neutrophils in another case (consistent with a diagnosis of acute ischemic cardiopathy) (Figure 4A). In one case (5%), abundant eosinophilic amyloid deposits were found in the interstitial tissue; positivity for Congo Red stain and an anti-transthyretin antibody (1/50, Quartett, Germany) was consistent with a diagnosis of senile amyloidosis (Figure 4B). In one case (5%), small foci of lymphocytes and macrophages were located close to myocardial fibers; this was consistent with a diagnosis of myocarditis (Figure 4C). Lastly, SARS-CoV-2 RNA was detected in 1 of the 6 patients tested; the patient’s myocardial tissue was normal, with no signs of myocarditis.

#### Liver Biopsies

All the liver biopsies (100%) were informative. Micronodular cirrhosis was diagnosed in 4 cases (20%) (Figure 4D). Hepatic blood vessel lesions were noted in 13 cases, with dilated centrilobular veins (13 cases), dilated sinusoids (13 cases), and fibrosis of perisinusoidal spaces (4 cases). Necrosis in the pericentrilobular spaces was seen in 2 cases. Mild steatosis (< 30%) was observed in 2 cases. Portal inflammation (with the presence of lymphocytes and macrophages) was seen in 6 cases. Lobular inflammation was seen in one case; it featured small granulomas comprising macrophages, lymphocytes, and a few eosinophils. A polymorphic inflammatory infiltrate was seen in the sinusoids in a few cases, and a large number of small lymphocytes were seen in one case. Vessel wall amyloid deposits were present in one case. Viral RNA was not detected in liver biopsies.

#### Muscle Biopsies

All the muscle biopsies (100%) were informative. The muscle tissue was normal, except for a few atrophic muscle fibers in some cases. No myositis was observed.

### Admission to the Intensive Care Unit

Compared with patients not admitted to the ICU, patients admitted to the ICU were younger (81.5 vs. 65.5 years of age, respectively; *p* = 0.01) and (because of life support provision) had a longer disease duration (16.5 vs. 33 days, respectively; *p* = 0.09) (Table 3). The time interval between disease onset and hospital admission was similar in the ICU and non-ICU groups (3.5 vs. 3 days, respectively; *p* = 0.82), as was the time interval between death and biopsy (20 h in both groups; *p* = 0.89). Patients admitted to the ICU had a greater prevalence of acute kidney damage [6 (75%), vs. 5 (41%) in the non-ICU group; *p* = 0.19] and myocardial damage; [5 (62.5%) vs. 3 (25%), respectively; *p* = 0.16].

**TABLE 3:** Patient characteristics as a function of ICU admission.

	Admitted to the ICU (*n* = 8)	Not admitted to the ICU (*n* = 12)	*p* value
Age	65.5 [61.8–75.3]	81.5 [72.8–87.8]	0.01
Sex: male/female ratio	7/1	7/5	0.32
Number of comorbidities, n	2 [1–2.25]	3 [2–4.5]	
Body mass index (kg/m^2^)	27.6 [25.8–29.9]	26.4 [23.2–29.1]	0.26
Time between disease onset and hospital admission (d)	3.5 [0.75–8.5]	3 [1.5–10]	0.82
Disease duration (time between disease onset and death) (d)	33 [19.5–48.8]	16.5 [9.3–24.5]	0.09
Time between death and biopsies (h)	20 [15–36]	20 [15.5–33.5]	0.89
Organ dysfunction			
Kidney damage	6 (75%)	5 (41%)	0.19
Myocardial damage	5 (62.5%)	3 (25%)	0.16
SARS-CoV-2 detected in the lung biopsy	4 (57.1%)	8 (72.7%)	0.62
Lung histopathological examination			
Diffuse/focal inflammatory lesions	5/3	3/8	0.18
Acute lesions	3 (37.5%)	9 (82%)	0.07
Sub-acute lesions	8 (100%)	10 (91%)	1
Fibrinous alveolitis	3 (37.5%)	2 (18.2%)	0.6
Macrophagic alveolitis	8 (100%)	11 (100%)	1
Neutrophilic alveolitis	0	7 (63.6%)	0.01
Organizing pneumonia with fibrous buds in the alveoli	6 (75%)	6 (54%)	0.69
Collapsed alveoli	6 (75%)	7 (63%)	0.35
Thrombosis	4 (50%)	6 (54%)	1
Dystrophic pneumocytes	7 (87.5%)	6 (54%)	0.17
Macrophages in the interalveolar septa	8 (100%)	9 (82%)	0.48
Lymphocytes in the interalveolar septa	5 (62.5%)	9 (82%)	0.60
Lymphocytic vasculitis	2 (25%)	6 (54%)	0.35

A pathology assessment of the lungs revealed greater prevalence of diffuse lesions, collapsed alveoli and dystrophic pneumocytes in the ICU group. Interestingly, acute lesions with neutrophilic alveolitis were observed more frequently in the non-ICU group (82%, vs. 37.5% in the ICU group for acute lesions; *p* = 0.07; 63.6% vs. 0%, respectively; *p* = 0.01, for neutrophilic alveolitis). In contrast, the ICU and non-ICU groups did not differ significantly with regard to the prevalence of sub-acute lesions (100% vs. 91%, respectively; *p* = 1), fibrinous alveolitis (37.5% vs. 18.5%, respectively; *p* = 0.6) and microvascular thrombosis. Lymphocytic infiltration and lymphocytic vasculitis were more prevalent in the non-ICU group, but the differences were not statistically significant.

### Differences in Pathology as a Function of the Disease Duration

We divided the cohort into 2 subgroups as a function of the median disease duration (Table 4). There were no intergroup differences in the proportion of patients admitted to the ICU (30% vs. 50% in the “early death” and “late death” groups, respectively; *p* = 0.65) or the proportion with viral RNA. The patients in the “early death” group had shorter disease duration and more focal inflammatory lesions in lungs. Conversely, patients in the “late death” group had a disease duration and a greater proportion of diffuse lesions (diffuse:focal ratio = 7:2, v. 1:9 in the “early death” group; *p* = 0.01). However, the groups did not differ with regard to the type of lesion (acute vs. sub-acute), the prevalence of neutrophilic, macrophagic and fibrinous alveolitis associated with collapsed alveoli, or the prevalence of macrophagic and lymphocytic infiltration in the interalveolar septa. Lymphocytic vasculitis was more prevalent in the “early death” group (60%, vs. 22.3% in the “late death” group but this difference was not statistically significant (*p* = 0.17).

**TABLE 4:** Pathology findings in the lung, by disease duration (early vs. late death).

	Early death (*n* = 10)	Late death (*n* = 10)	*p* value
Admission to the ICU	3 (30%)	5 (50%)	0.65
SARS-CoV-2 detected in the lung biopsy	6 (60%)	7 (70%)	1
Histopathological assessment of the lungs:	*n* = 10	*n* = 9	
Diffuse/focal inflammatory lesions	1/9	7/2	0.01
Acute lesions	6 (60%)	6 (66.7%)	1
Subacute lesions	10 (100%)	8 (88.9%)	0.47
Fibrinous alveolitis	2 (20%)	3 (33.4%)	0.63
Macrophagic alveolitis	10 (100%)	9 (100%)	1
Neutrophilic alveolitis	4 (40%)	3 (33.4%)	1
Organizing pneumonia with fibrous buds in the alveoli	5 (50%)	7 (77.8%)	0.35
Collapsed alveoli	7 (70%)	6 (66.7%)	1
Dystrophic pneumocytes	6 (60%)	7 (77.8%)	0.63
Macrophages in interalveolar septa	7 (70%)	5 (55.6%)	0.65
Lymphocytes in interalveolar septa	10 (100%)	8 (88.9%)	0.47
Lymphocytic vasculitis	6 (60%)	2 (22.3%)	0.17
Thrombosis	5 (50%)	7 (77.8%)	0.35

### Causes of Death, Based on the Pathology Findings

Based on the pathology findings, a cause of death was determined in 19 cases (95%). Pneumonitis was found in all 19 patients (100%), with aspiration pneumonitis in 2 patients (10.5%) and acute bronchopneumonia (due to a clinically suspected bacterial infection) in one (5.2%). Cardiac decompensation was diagnosed in 11 cases (58%) and was variously related to ischemic cardiomyopathy (*n* = 9; 47%), cardiac amyloidosis (*n* = 1; 5.2%), and myocarditis (*n* = 1; 5.2%). Pulmonary embolism was the suspected cause of death in 1 patient (5.2%). Acute pyelonephritis (due to a suspected bacterial infection but in the absence of microbiological test data) was found in 1 patient (5.2%).

Acute decompensation of chronic disease was diagnosed for 14 patients (73.7%), with cardiac decompensation in 10 (52.6%) and acute on Chronic Liver Failure in 4 (21%).

Correlation between clinical data with organ failure and pathological findings are resumed in the Supplementary Table.

## Discussion

We described post-mortem multi-organ biopsies in fatal cases of COVID-19. Our results not only confirmed the major role of lung damage but also highlighted the potential involvement of extra pulmonary disease.

The main histological findings in lung biopsies from patients who died after a confirmed SARS-CoV-2 infection were focal, patchy, organizing pneumonia with collapsed alveoli, thickened alveolar walls (containing lymphocytic infiltrates and a few macrophages), and fibrous buds in the bronchiolar and alveolar spaces. These observations were in line with the literature data. Macrophage and multinuclear cell infiltration in alveolar spaces and desquamated hyperplastic pneumocytes with enlarged nuclei have also been described previously.

Conversely, (i) diffuse alveolar damage with hyaline membranes in all the alveoli and (ii) fibrin balls (as seen in acute fibrinous organizing pneumonia) were rarely observed; this contrasts with the literature data (22–25). Although endothelial injury with cytoplasmic vacuolization and cell detachment in pulmonary arteries or megakaryocytes in alveolar capillaries has been described previously, we did not observe any such patterns in the present study (23, 25). It is important to notice that viral RNA was detected in ICU patients even after long disease duration. The TEM ultrastructure analysis showed viral particles after 17 and 27 days of illness. This illustrates a prolonged active viral replication in lungs. Interestingly, the prolonged viral shedding was not associated with prior immunodeficiency or immunosuppressive treatment in our cohort. It has been shown that patients with severe COVID-19 impaired the ability to contain viral replication by an insufficient humoral anti SARS-CoV-2 response and/or insufficient type 1 interferon amplification (26, 27).

Interestingly, these COVID-19-associated histopathological profiles are not specific and are seen in the ARDS induced by H1N1 influenza and other viruses. However, the proportion of our cases with DAD was lower than in other histopathological studies of COVID-19 and the same as in a large study of cases of ARDS (28). Diffuse alveolar damage was more prevalent in our ICU group than in our non-ICU group.

There are several possible explanations for these differences. Firstly, life support in the ICU was associated with longer disease duration; this may have given more time for lesions to spread. Indeed, we often observe in the most severe cases, an extension of the lesions on computed tomographic imaging reassessment. Secondly, all the patients in the ICU group received mechanical ventilation while non-ICU patients only received standard oxygen therapy. Therefore, ICU patients might have developed ventilator-induced lung injuries (VILI). VILI is ascribed to volutrauma, barotrauma and atelectrauma and contribute to mortality in patients with ARDS. These phenomenon can be associated with self-inflicted lung injury in ventilated patients (29). In addition, ventilator-associated pneumonia can occur in these patients and contribute to lung inflammation. These mechanisms are likely to aggravate lung damage in COVID-19 and might explain the differences observed in our study. The observed differences were unlikely to be related to the time interval between death and biopsy, which was similar in the ICU and non-ICU groups.

The biopsies of organs other than the lungs were also informative. Myocardial damage is reportedly common in COVID-19, but we did not find specific evidence of this in our cohort. Most patients displayed cardiac decompensation associated with chronic ischemic cardiomyopathy or cardiac amyloidosis. Myocarditis was found in one patient only, and SARS-CoV-2 RNA was detected in another patient. These findings suggest that comorbidities have an important influence on the clinical outcome in patients with an acute SARS-CoV-2 infection. Interestingly, hepatic blood vessel lesions were prevalent among our cases, with dilatation of centrilobular veins, dilatation of sinusoids, and fibrosis of perisinusoidal spaces. The liver damage might therefore have been related to decompensated heart failure, rather than COVID-19 *per se*.

In our TEM ultrastructural analysis of lung biopsies, viral particles were observed in only 3 of the 6 patients—despite our careful examination of lung cells and the presence of the viral genome in an RT-PCR assay. This finding emphasized the low sensitivity of TEM-based SARS-CoV-2 particle detection for post-mortem diagnosis. Ultrathin (100 nm) sections do not give an overview of the cellular and tissue volumes. Delayed post-mortem fixation might also change the enveloped particles’ structure and thus make them hard to identify. The levels of viral particle replication and assembly change over the course of COVID-19 and (in late-stage infections) depend on the host response. Furthermore, the recently described potential for misidentifying clathrin-coated vesicles or nuclear pores as viral particles means that the TEM-based identification of coronaviruses should be considered with caution (19–21).

However, one of our study’s major findings was the TEM-based identification of host-cell membrane rearrangements (DMVs) characteristic of SARS-CoV-2 infection in kidney tissues in two patients. Furthermore, the presence of DMVs in tubular epithelial cells evidenced intense viral replication in the kidney. To the best of our knowledge, our study has provided the first description of DMVs in a biopsy from a patient infected by SARS-CoV-2; previously, these structures have only been observed for infected cells cultured *in vitro*. In the absence of viral particles in the cytoplasm, this specific marker of viral replication might be very useful for identifying SARS-CoV-2-infected cells in a tissue sample.

Surprisingly, DMVs were observed in the kidneys but not in the lungs. Furthermore, viral particles were observed not only in renal endothelial cells but also in podocytes and tubular epithelial cells; this observation is consistent with previous histopathological findings (30, 31). These features highlighted the multiorgan tropism of SARS-CoV-2, which can infect and replicate in various cell types outside the respiratory tract.

The present study had a number of limitations. Firstly, we performed post-mortem needle and surgical biopsies rather than a full, conventional autopsy. No accidental viral contaminations were observed during the study period (data not shown). We conclude that post-mortem needle and surgical biopsies constitute a safe procedure that enables clinical correlations and assessment of the cause of death in most cases. Secondly, we performed TEM on biopsies from a small number of patients and organs. Even though our findings were in line with the literature data, the small number of samples prevented us from drawing any definitive conclusions with regard to the tissue ultrastructure.

## Conclusion

Several differences were observed between ICU and non-ICU patients regarding lung damage. Our present results also highlighted the involvement of damage outside the lung in the fatal progression of COVID-19. Cardiac and hepatic damage was probably related to chronic disease decompensation, whereas kidney injury was more likely to be related to viral dissemination. Our TEM study was the first to describe the presence of virus-induced DMVs in kidney biopsies and thus provided unequivocal proof of intense SARS-CoV-2 replication in this organ. When seeking to describe lesions and identify the cause of death in cases of COVID-19, needle and surgical post-mortem biopsies are good surrogates for conventional autopsies.

## Data Availability Statement

The raw data supporting the conclusions of this article will be made available by the authors, without undue reservation.

## Ethics Statement

The studies involving human participants were reviewed and approved by French Ministry of Health. Written informed consent for participation was not required for this study in accordance with the national legislation and the institutional requirements.

## Author Contributions

YZ, CB, and MS collected data. DC, NG, SE, EB, RU, and PR performed the pathological assessments. YZ, DC, SE, RU, SC, EB, and PR wrote the manuscript. All authors critically reviewed the manuscript and approved the final version of the manuscript.

## Conflict of Interest

The authors declare that the research was conducted in the absence of any commercial or financial relationships that could be construed as a potential conflict of interest.

## Publisher’s Note

All claims expressed in this article are solely those of the authors and do not necessarily represent those of their affiliated organizations, or those of the publisher, the editors and the reviewers. Any product that may be evaluated in this article, or claim that may be made by its manufacturer, is not guaranteed or endorsed by the publisher.
