# Supplementary material for: Pathology Assessments of Multiple Organs in Fatal COVID-19 in Intensive Care Unit vs. Non-intensive Care Unit Patients
Source: Front Med (Lausanne). 2022 Apr 25;9:837258. doi: 10.3389/fmed.2022.837258 (PMC9081791; doi:10.3389/fmed.2022.837258)
Supplement: Supplementary file 1 [file Table_1.DOCX]

**Supplementary table**

|  | Ageyears | **Clinical findings** | Pathological findings | | | |
| --- | --- | --- | --- | --- | --- | --- |
|  |  |  | Lung | Kidney | Myocardium | Liver |
| **11 1** | 66 | Not admitted to ICU, extend lung lesions, No AKI, AHF, cholestasis | Not informative | Normal | Interstitial fibrosis | ACLI |
| **2** | 73 | Not admitted to ICU, hypoxemic ARF,  no AKI,  icteric cholestasis | Focal, chronic fibro-inflammatory lesions, Interstitial organized pneumopathy, Dystrophic vessels, Mild alveolitis | Not informative | Focal myocarditis | Congestive hepatopathy |
| **3** | 83 | Not admitted to ICU, hypoxemic ARF, AKI KDIGO 2, global AHF, hepatic cytolysis and cholestasis | Focal, chronic fibro-inflammatory lesions, Interstitial organized pneumopathy, Fibrinous thrombi, Dystrophic vessels, Mild alveolitis | FSGH, atherosclerosis and unspecific nephritis | Amyloidosis | ACLI,  Necrosis in the pericentrilobular spaces, Amyloidosis |
| **4** | 93 | Not admitted to ICU, hypoxemic ARF, extend lung lesions, CKI no AKI, no AHF, no ALF | Diffuse, acute lesions, Interstitial organized pneumopathy, Fibrinous thrombi, Dystrophic vessels, Alveolitis | Normal | Normal | Mild portal fibrosis and inflammation |
| **5** | 78 | Not admitted to ICU, hypoxemic ARF, No sign of AHF or ALF | Diffuse, acute lesions, Interstitial organized pneumopathy, Lymphocytic vasculitis, Alveolitis | Not informative | Interstitial fibrosis | ACLI |
| **6** | 87 | Not admitted to ICU, hypoxemic ARF, AKI KDIGO 2, right-sided AHF, ALF | Focal, chronic fibro-inflammatory lesions, Interstitial organized pneumopathy, Fibrinous thrombi, Lymphocytic vasculitis, Dystrophic vessels, Alveolitis | Not informative | Interstitial fibrosis, acute myocardial infarction remodeling | ACLI, Necrosis in the pericentrilobular spaces |
| **7** | 90 | Not admitted to ICU, hypoxemic ARF, AKI KDIGO 2, No sign of AHF or ALF | Focal, chronic fibro-inflammatory lesions, Interstitial organized pneumopathy, Lymphocytic vasculitis, Alveolitis | Not informative | Normal | Mild portal fibrosis and inflammation |
| **8** | 62 | Severe ARDS, low rs compliance, requiring VV-ECMO, septic shock, AKI KDIGO 3, right-sided AHF, ALF | Focal, acute and chronic fibro-inflammatory lesions, Interstitial organized pneumopathy, Fibrinous thrombi, Lymphocytic vasculitis, Dystrophic vessels, Alveolitis | Not informative | Interstitial fibrosis | Mild ACLI, Congestive hepatopathy |
| **9** | 71 | Not admitted to ICU, hypoxemic ARF, AKI KDIGO 3, No sign of AHF or ALF | Diffuse, acute lesions, Interstitial organized pneumopathy, fibrinous thrombi, Lymphocytic vasculitis, Dystrophic vessels, Mild alveolitis | Not informative | Normal | Moderate portal fibrosis and inflammation |
| **10** | 59 | Moderate ARDS, No AKI, Decompensated cirrhosis, No AHF, | Focal, acute and chronic fibro-inflammatory lesions, Interstitial organized pneumopathy, Alveolitis | Atherosclerosis | Interstitial fibrosis | Cirrhosis |
| **11** | 72 | Not admitted to ICU, hypoxemic ARF, No AHF, hepatic cytolysis and cholestasis | Focal, chronic fibro-inflammatory lesions, Interstitial organized pneumopathy, Lymphocytic vasculitis, Alveolitis | Atherosclerosis, interstitial nephritis | Normal | Portal inflammation, Moderate steatosis |
| **12** | 67 | Severe ARDS, septic shock, AKI KDIGO 3, No sign of AHF or ALF | Diffuse, acute lesions, Interstitial organized pneumopathy, fibrinous thrombi, Dystrophic vessels, Alveolitis | Not informative | Normal | Cirrhosis |
| **13** | 82 | Not admitted to ICU, hypoxemic ARF, AKI KDIGO 3, left-sided AHF and pulmonary hypertension, hepatic cytolysis | Focal, chronic fibro-inflammatory lesions, Interstitial organized pneumopathy, Lymphocytic vasculitis, Alveolitis | Atherosclerosis, interstitial nephritis | Normal | Portal inflammation, Focal ACLI |
| **14** | 84 | Not admitted to ICU, hypoxemic ARF, AKI KDIGO 2, No sign of AHF or ALF | Diffuse, acute and chronic fibro-inflammatory lesions, severe fibrinous thrombi, Alveolitis | FSGH, atherosclerosis and unspecific nephritis | Interstitial fibrosis | ACLI, mild steatosis |
| **15** | 85 | Not admitted to ICU, hypoxemic ARF, AKI KDIGO 3, right-sided AHF, hepatic cytolysis and cholestasis | Focal, acute and chronic fibro-inflammatory lesions, Interstitial organized pneumopathy, mild fibrinous thrombi, Dystrophic vessels, Alveolitis | Atherosclerosis and unspecific nephritis | Interstitial fibrosis, fibrinous pericarditis | ACLI, Congestive hepatopathy, severe fibrosis of perisinusoidal spaces |
| **16** | 64 | Severe ARDS requiring VV-ECMO, AKI KDIGO 3, right-sided AHF, cholestasis | Diffuse, acute lesions, Interstitial organized pneumopathy, Dystrophic vessels, Mild alveolitis | Normal | Normal | Cirrhosis with lymphocytic infiltrate |
| **17** | 93 | Not admitted to ICU, hypoxemic ARF, no AKI, no sign of AHF or ALF | Focal, acute and chronic fibro-inflammatory lesions, Interstitial organized pneumopathy, fibrinous thrombi, Lymphocytic vasculitis, Dystrophic vessels, Mild alveolitis | Not informative | Interstitial fibrosis | ACLI, Congestive hepatopathy |
| **18** | 61 | Severe ARDS requiring VV-ECMO, AKI KDIGO 3, AHF, hepatic cytolysis and cholestasis | Diffuse, acute and chronic fibro-inflammatory lesions, Interstitial organized pneumopathy, fibrinous thrombi, Dystrophic vessels, Alveolitis | Normal with post mortem necrosis | Interstitial fibrosis and neutrophils infiltration | Congestive hepatopathy |
| **19** | 80 | Hypoxemic ARF and cardiac arrest. Shock and AHF, no ALF | Focal, acute and chronic fibro-inflammatory lesions, Interstitial organized pneumopathy, fibrinous thrombi, Dystrophic vessels, Alveolitis | Acute pyleonephritis | Normal | ACLI, Congestive hepatopathy |
| **20** | 73 | Severe ARDS requiring VV-ECMO, septic shock, AKI KDIGO 2, hepatic cytolysis and cholestasis | Diffuse, acute lesions, Interstitial organized pneumopathy, Alveolitis | Mild FSGH, mild atherosclerosis and unspecific nephritis | Normal | ACLI |

ACLI denotes acute cardiogenic liver injury, AHF denotes acute heart failure, AKI denotes acute kidney Injury, ARDS denotes acute respiratory distress syndrome, ARF denotes acute respiratory failure, CKI denotes chronic kidney insufficiency, FSGH denotes focal segmentary glomerular hyalinosis, VV-ECMO denotes veno-venous extracorporeal membrane oxygenation, rs denotes respiratory compliance.
